# Emotional modulation of synapses, circuits and memory

**DOI:** 10.3389/fnbeh.2015.00035

**Published:** 2015-02-19

**Authors:** Jonathan E. Ploski, Christa K. McIntyre

**Affiliations:** Cognition and Neuroscience Program, School of Behavioral and Brain Sciences, The University of Texas at DallasRichardson, TX, USA

**Keywords:** amygdala, fear conditioning, extinction, reconsolidation, oscillations, synapse allocation

Emotion is a powerful tool for change in the central nervous system. Whereas, most long-term memories are stored after practice or rehearsal, an emotionally arousing memory can be consolidated after a single experience. Research examining the influence of emotion on synaptic function provides a window of opportunity for exploring the mechanisms of memory consolidation during the minutes to hours after a single, well-remembered experience. It can also shed light on numerous psychiatric conditions, bringing the field closer to preventing or treating those conditions that stem from traumatic memories. This research topic brings together leading experts who share their recent findings and perspectives on how emotion may influence brain function.

In this issue, four articles review or describe evidence of a specialized influence of emotion on synaptic function. Sheena Josselyn's laboratory previously found that neurons with high levels of cAMP Responsive Element Binding Protein (CREB) at the time of training are preferentially allocated to the memory trace (Han et al., 2007). In this issue, Sargin et al. (2013) provide a cellular mechanism as to why this may be. They report that overexpression of CREB within neurons of the lateral nucleus of the amygdala (LA), leads to an increase in dendritic spine density, whereas LA neurons with low CREB activity exhibit a decrease in dendritic spine density. These data support the hypothesis that CREB may increase a neuron's propensity for being included in a memory trace by increasing dendritic spine density.

Young and Williams addressed the issue of lateralization of amygdala recruitment during memory consolidation. Imaging studies in humans indicate that activity in the right amygdala is associated with aversive memories (Morris et al., 1999). Others indicate that the left amygdala is preferentially involved in encoding memories that have a positive valence (Zalla et al., 2000; Hamann et al., 2002). In this issue, Young and Williams (2013) examine expression of a marker of synaptic plasticity in the rat amygdala. They report that the synaptic plasticity-related protein Arc is specifically elevated in the right amygdala following training on an aversive task, and in the left amygdala following training on an appetitive task. Considering that Arc expression in the amygdala is necessary for consolidation of conditioned fear (Ploski et al., 2008), these findings indicate that, memory-related synaptic plasticity in the amygdala is lateralized and valence-dependent.

Headley and Paré (2013) add a temporal dimension to this research topic. They review literature on temporal synchrony of firing across brain regions involved in emotional memory. They describe gamma oscillations and emotional memory, citing recent reports of enhanced gamma oscillations in the neocortex and amygdala during emotional situations, and evidence that gamma oscillations have predictive value for synaptic plasticity and emotional memory.

Grønli et al. (2013) take a different view on the effects of emotion on memory and plasticity. These authors review the cellular and molecular evidence that stress may impair cognition by interfering with sleep. They describe the importance of sleep for the cellular and molecular processes that contribute to memory consolidation, and suggest that stress that interferes with sleep is associated with deficient synaptic plasticity and impaired cognitive performance.

Several of the contributions to this issue examined synaptic changes related to extinction of conditioned fear in rats. Both post-traumatic stress disorder (PTSD) and obsessive compulsive disorder (OCD) are characterized by avoidance of stimuli that are perceived as threatening, even in the absence of real danger. Impaired extinction of conditioned fear could contribute to the persistence of maladaptive behaviors seen in these disorders (Milad et al., 2008, 2013). It was recently demonstrated that vagus nerve stimulation (VNS) enhances extinction of conditioned fear in rats (Peña et al., 2013). In this issue, (Peña et al., 2014) report that extinction-enhancing VNS reverses synaptic depression in the infralimbic prefrontal cortex basolateral amygdala pathway, a circuit that is implicated in extinction memory (Sierra-Mercado et al., 2011). Collectively, these findings suggest that VNS could be paired with exposure therapy to facilitate extinction of conditioned fear and reverse pathological synaptic function seen in anxiety disorders. Another contribution investigated the effects of deep brain stimulation (DBS) on the extinction circuitry. In 2010, several groups reported that stimulation of the ventral capsule/ventral striatum reduced symptoms of OCD in refractory patients (Denys et al., 2010; Goodman et al., 2010; Greenberg et al., 2010). In rats, stimulation of the ventral striatum enhances extinction of conditioned fear (Rodriguez-Romaguera et al., 2012). In this issue, research from Greg Quirk's lab demonstrates that extinction-enhancing stimulation of the ventral striatum increases expression of the brain-derived neurotrophic factor protein (BDNF) in the medial prefrontal cortex, indicating that the clinical benefits of DBS may be mediated by BDNF-associated synaptic changes in the extinction pathway (Do-Monte et al., 2013).

One of the limitations of exposure therapy is that conditioned fear can return even after successful extinction learning. In 2009, Monfils and colleagues reported evidence that pairing of a brief retrieval trial with extinction training produced a persistent reduction of conditioned fear that was not susceptible to spontaneous recovery, reinstatement, or renewal of fear (Monfils et al., 2009). This effect is now referred to as “reconsolidation updating” because the brief retrieval trial is thought to destabilize the memory trace. The destabilized conditioned fear memory trace is thought to be modified by the extinction training. However, because some laboratories do not observe persistent reductions in fear using the reconsolidation-extinction approach, researchers in the laboratories of Marie Monfils and Hongjo Lee collaborated to determine whether orienting phenotype affects the persistence of the fear reduction. These authors contributed two articles to this issue. Both of these studies distinguished rats that direct attention to a conditioned stimulus during appetitive conditioning (orienters) from rats that do not (non-orienters). Expression of conditioned fear 24 h after training is greater in non-orienters. However, pairing a brief retrieval trial with an extinction session prevents spontaneous recovery of fear in both phenotypes, indicating that orienting phenotype is not a boundary condition that would interfere with the permanence of the effect (Olshavsky et al., 2013a). In contrast, orienting phenotype has a significant effect on the persistence of reconsolidation updating when the conditioned stimulus is appetitive. Conditioned responding spontaneously recovers only in the non-orienting phenotype (Olshavsky et al., 2013b).

Historically, reconsolidation updating and extinction learning have been considered separate processes. However, with the discovery and development of the reconsolidation-extinction paradigm (Monfils et al., 2009), it has become apparent that there is overlap between reconsolidation and extinction processes. In this issue Flavell and colleagues review what is known about the cellular and molecular mechanisms that govern the reactivation-dependent destabilization of memory and how these processes influence the permanent weakening of memory. Considering not all memories become destabilized upon retrieval, and therefore are resistant to being modified, this area of research holds great promise for the development of treatments targeting pathological memory (Flavell et al., 2013).

Roesler and colleagues sought to enhance extinction of conditioned fear in rats but instead found that intra-dorsal hippocampus infusions of the phosphodiesterase type 4 (PDE4) inhibitor, rolipram, during extinction training, can switch the behavioral outcome from extinction to enhanced fear (Roesler et al., 2014). These intriguing findings indicate that inhibition of PDE4 during extinction training may promote reconsolidation update mechanisms and/or inhibit extinction learning.

Extensive evidence supports the role of *de novo* protein synthesis in the consolidation and reconsolidation of memory. In addition, there is accumulating evidence indicating that coordinated protein degradation via the ubiquitin-proteasome system (UPS) is required for these processes. For example, inhibition of the UPS disrupts the consolidation of memory and the destabilization phase of memory updating (Jarome and Helmstetter, 2013). Recently Kwapis et al. (2014) demonstrated that protein degradation via the UPS, within the LA was critical for the consolidation of delay fear conditioning. In this issue, Reis et al. (2013) extend these findings by demonstrating that pharmacologically inhibiting the UPS, with the proteasome inhibitor clasto-lactacystin-β-lactone (β-lac) within the prefrontal cortex, selectively disrupts trace fear conditioning, while leaving delay fear conditioning intact. These findings further support the role of the PFC in trace, but not delay conditioning and underscore the wide spread importance of UPS mediated protein degradation in learning and memory phenomena.

Animal models of trauma and anxiety disorders are helpful in translating these discoveries to therapies. Berardi et al. (2014) present a rodent model of PTSD that meets cognitive and emotional criteria for diagnosis according to the most recent version of the Diagnostic and Statistical Manual of Mental Disorders (DSM-5). They found that rats exposed to inescapable footshocks and housed in isolation exhibited fear of the context that was associated with the shock for 56 days. These rats also exhibited long-lasting alterations in social interactions and exploration on the elevated plus maze. Such advancements in animal models of PTSD may provide new avenues for exploring the effects of traumatic experiences on the brain as well as opportunities for testing possible therapies.

## Conflict of interest statement

The authors declare that the research was conducted in the absence of any commercial or financial relationships that could be construed as a potential conflict of interest.
